# Unravelling the effect of New Year’s Eve celebrations on SARS-CoV-2 transmission

**DOI:** 10.1038/s41598-023-49678-x

**Published:** 2023-12-14

**Authors:** Caspar Geenen, Jonathan Thibaut, Lies Laenen, Joren Raymenants, Lize Cuypers, Piet Maes, Simon Dellicour, Emmanuel André

**Affiliations:** 1 Department of Microbiology, Immunology and Transplantation, Laboratory of Clinical Microbiology, KU Leuven, Herestraat 49, 3000 Leuven, Belgium; 2 Department of Laboratory Medicine, National Reference Centre for Respiratory Pathogens, University Hospitals Leuven, Leuven, Belgium; 3 Rega Institute, Department of Microbiology, Immunology and Transplantation, Laboratory of Clinical and Epidemiological Virology, KU Leuven, Leuven, Belgium; 4 Spatial Epidemiology Lab, Université Libre de Bruxelles, Brussels, Belgium

**Keywords:** Viral infection, SARS-CoV-2, Viral epidemiology

## Abstract

Public holidays have been associated with SARS-CoV-2 incidence surges, although a firm link remains to be established. This association is sometimes attributed to events where transmissions occur at a disproportionately high rate, known as superspreading events. Here, we describe a sudden surge in new cases with the Omicron BA.1 strain amongst higher education students in Belgium. Contact tracers classed most of these cases as likely or possibly infected on New Year's Eve, indicating a direct trigger by New Year celebrations. Using a combination of contact tracing and phylogenetic data, we show the limited role of superspreading events in this surge. Finally, the numerous simultaneous transmissions allowed a unique opportunity to determine the distribution of incubation periods of the Omicron strain. Overall, our results indicate that, even under social restrictions, a surge in transmissibility of SARS-CoV-2 can occur when holiday celebrations result in small social gatherings attended simultaneously and communitywide.

## Background

Surges in COVID-19 case numbers have been observed following public holidays and celebrations, although causal evidence remains limited^1–7^. Modelling studies and empirical data have implicated superspreading events (SSE) as major contributors to the spread of SARS-CoV-2 and other pathogens^8–12^. A SSE is an event where a disproportionately high number of transmissions take place. The exact role of SSEs in holiday-triggered SARS-CoV-2 incidence surges remains unclear.

Here, we investigate the effect of New Year’s Eve celebrations in Belgium on the incidence of SARS-CoV-2, specifically within a local community of higher education students. Anticipating a rise in cases due to the emerging B.1.1.529 lineage – corresponding to the Omicron variant of concern – Belgian authorities implemented increasingly stringent restrictions on social interactions in November and December 2021. By the end of December, indoor mass gatherings were prohibited, face masks were required in public indoor areas and working from home was mandatory in many professional sectors. Schools were closed for the winter holidays from 25 December 2021 until 9 January 2022. Christmas and New Year celebrations were essentially limited to private gatherings in residential locations, although no limit was imposed on the number of attendees. Despite these measures, a surge in COVID-19 cases was detected after New Year’s Eve 2022 (NYE) by our targeted test and trace program for higher education students in the city of Leuven^13^.

Estimates of the median and mean incubation periods have been reported for several variants of concern and are generally lower than for the ancestral SARS-CoV-2 strain^14^. However, accurate assessment of the shape of the incubation period distribution is, in practice, much harder, because it requires a high level of certainty about the moment of inoculation for a sufficiently large sample of cases^15^. Contact tracing can sometimes provide such information, by tracing individuals exposed to an infectious case (forward contact tracing), or to the suspected source case or event (backward contact tracing or source investigation). In this way, Tanaka et al. previously observed a mean incubation period of 3.03 days (SD: 1.35) for the Omicron BA.1 lineage^16^.

Using a combination of phylogenetic and contact tracing data, we aimed to investigate the relationship between NYE celebrations and Omicron case numbers in a population of higher education students in the Belgian city of Leuven. We also use the resulting dataset to determine the distribution of incubation periods of the Omicron BA.1 strain in this population.

## Results

### Epidemic curve

We focused our analyses on the Omicron BA.1 strain, which became dominant over the Delta strain amongst students in Leuven in mid-December 2021 (Supplementary Fig. 1). Vaccination rates in this population were above 90% during the study period, with a mean of three months elapsed since the last dose (Supplementary Fig. 5).

**Figure 1:**
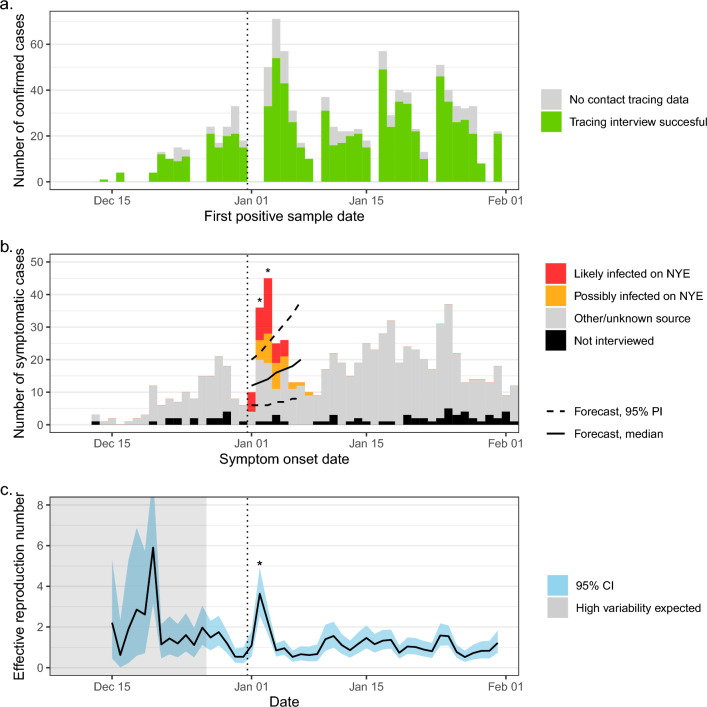
Evolution of Omicron BA.1 case numbers amongst higher education students at KU Leuven, Belgium. Panel a shows daily totals of Omicron BA.1 cases detected by the university test centre, stratified by sampling date. Successfully interviewed cases are shown in green. In panel b, symptomatic cases are shown by self-reported symptom onset date. Classifications of “possibly infected on NYE” (New Year's Eve 2022) in yellow and “likely infected on NYE” in red were assessed by a contact tracer as described in the Methods section. Solid and dashed black lines indicate the median and 95% prediction interval (PI), respectively, of a 7-day forecast based only on case numbers before NYE. Asterisks indicate observations outside the 95% PI of the forecast. Panel c shows one-day sliding window estimates of the effective reproduction number (R_eff_) for the student population in Leuven, derived from the epidemic curve in panel b using EpiEstim^17^. The blue band indicates the 95% credible interval (CI), while the grey area indicates the period when incidence was too low to reach an expected coefficient of variation above 0.3. An asterisk indicates that a significant outlier was detected using a one-sided Grubbs test. Vertical dotted black lines indicate NYE. In each panel, the graphs show only PCR-confirmed cases with a negative S-gene target on the TaqPath COVID-19 assay, corresponding to the Omicron BA.1 strain.

Our test and trace program for the Leuven student population detected a short-lived surge in cases at the start of 2022, after winter holiday celebrations. Although the surge was associated with a rise in the number of PCR tests performed at the test centre, the test positivity rate did not decrease (Supplementary Fig. 1). In a target population of around 50,000 higher education students, 2,505 underwent a test in the first two weeks of the new year, of which 424 (16.9%) had a positive result. Twelve cases were excluded because the S-gene target of the PCR assay was positive and 35 because the signal was too weak (C_q_ value for N-gene target above 30) to confirm or exclude S-gene target failure. Another 13 were excluded because at the time of sampling they already had a recent diagnosis of COVID-19. Of the remaining cases, 286 (79%) were successfully interviewed by the contact tracing team and grouped according to the criteria described in the *Methods* section: possibly infected on NYE (36 cases, 13%), likely infected on NYE (54 cases, 19%), or other/unknown source (196 cases, 69%). Symptomatic cases were plotted by onset date to construct the epidemic curve (Fig. 1).

At the peak of the epidemic curve on 3 January 2022, those likely and possibly infected on NYE accounted for 59% of all symptomatic cases (26 cases out of 44), of which 65% (17 cases) were classed as likely infected on NYE.

### Effective reproduction number

We estimated daily values of the reproduction number (R_eff_) from the epidemic curve, using the method by Cori et al. with a one-day sliding window (Fig. 1*, panel c*)^17^. R_eff_ reached a peak of 3.6 (95% CI: 2.6–4.9) two days after the suspected peak in transmissions on NYE. This is consistent with a lag inherent to this method, approximately equal to the incubation period^17^. The peak and the lower bound of its credible interval were confirmed as significant outliers using a Grubbs test (p = 0.00001 and p = 0.015, respectively).

With the same method to estimate R_eff_, but using only case numbers before NYE as input, we estimated a forecast for the subsequent 7 days (Fig. 1, panel b). Actual case numbers rose considerably above the 95% prediction interval on 2 and 3 January, but not when excluding cases likely or possibly infected on NYE.

### Case characteristics

The mean age of the 90 cases possibly or likely infected on NYE was 21.7 years (SD: 2.2 days) and 51% of cases were female. The vaccination rate amongst these cases was 95% (82 of 86 cases; missing data: 4 cases) and, for vaccinated individuals, the mean number of months elapsed since the last dose was 3.3 (95% CI: 2.9–3.7). The mean follow-up period of the 15 asymptomatic cases was 4.5 days after sampling of the positive test (range: one to nine days). Some cases (26 cases, 29%) attended a shared NYE event with one or more other cases possibly or likely infected on NYE. However, no single event was identified linking more than four cases.

Before our phylogenetic analysis, we used information from the contact tracing interviews to assess the role of superspreading events. 17 cases (19%) declined to share the total number of attendees at their NYE event. Out of the remaining cases, 30 (59%) reported being infected at an event with fewer than ten attendees. That means a superspreading event could be excluded, based on our definition of nine or more transmissions (see the *Methods* section for more detail). We also asked each case directly about known test results of other attendees, which included persons outside the region and scope of our contact tracing efforts. Negative test results thus excluded a superspreading source event for 48 cases (66%), whereas twelve cases (16%) originating at a SSE were infected at five separate events. Thus, the share of transmissions originating from a SSE was situated in the range of 16 to 34%.

### Phylogenetic analysis

We performed a phylogenetic analysis to further clarify whether a small number of SSEs could have led to the observed surge in infections.

Out of the 90 cases possibly or likely infected on NYE, viral genomic sequencing was not attempted for five due to a weak positive PCR result. Whole genome sequencing failed for two samples, resulting in 83 successfully determined genomes (92%). A time-scaled phylogeny was constructed based on (*a*) these 83 sequences, (*b*) all 7,933 Belgian sequences belonging to the Omicron strain available on GISAID, collected between 1 November 2021 and 20 January 2022 and analysed for the purpose of baseline surveillance, and (*c*) a subset of 753 Omicron sequences collected during the same period in other European countries (see the *Methods* section for more detail)^18^.

We excluded 397 sequences, including 3 obtained from NYE samples, because they did not belong to the BA.1 lineage. Another 254 sequences, including one NYE sample, were excluded because the length was shorter than 27,000 bases. The geographic spread of all included samples is shown in Supplementary Fig. 4. The most common Pango lineages were BA.1.1 (20 cases), BA.1 (18 cases), BA.1.17.2 (13 cases), BA.1.14 (10 cases), and BA.1.17 (8 cases). Other lineages are shown in Supplementary Table 5.

**Figure 2:**
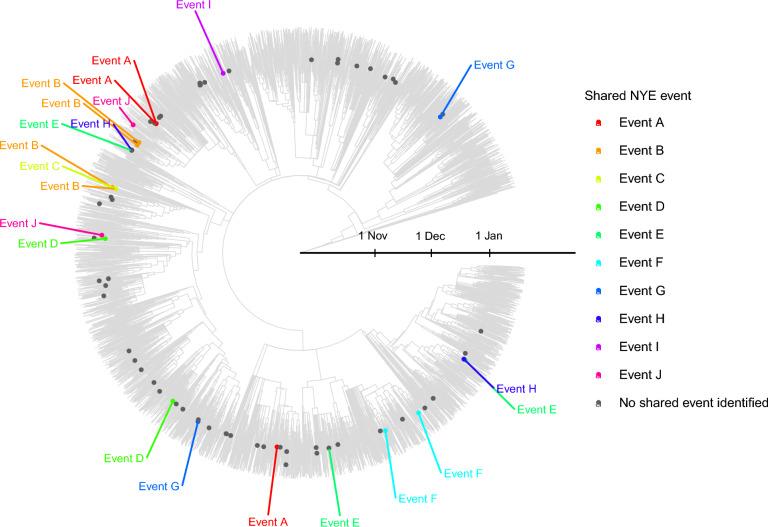
Phylogenetic context of NYE related infections in the student population. This figure shows a time-scaled phylogeny based on the maximum likelihood phylogenetic analysis of an alignment including all Belgian baseline surveillance sequences of the Omicron BA.1 lineage available on GISAID and sampled until 20 January 2022, as well as a selection of BA.1 sequences from other European countries. Dots show sequences obtained from Leuven students possibly or likely infected on NYE, coloured by which shared event they attended.

In the resulting phylogenetic tree of 8,118 sequences, the NYE sequences spanned the range of strains circulating on a national scale (Fig. 2). Out of all 79 included sequences linked to NYE, 29 (37%) clustered together with one to three other NYE sequences, when using the cov2clusters clustering method^19^. However, most appeared isolated from other NYE sequences in the overall tree. No more than four NYE sequences could be grouped with 90% probability in a single cluster .

### Incubation period

**Figure 3:**
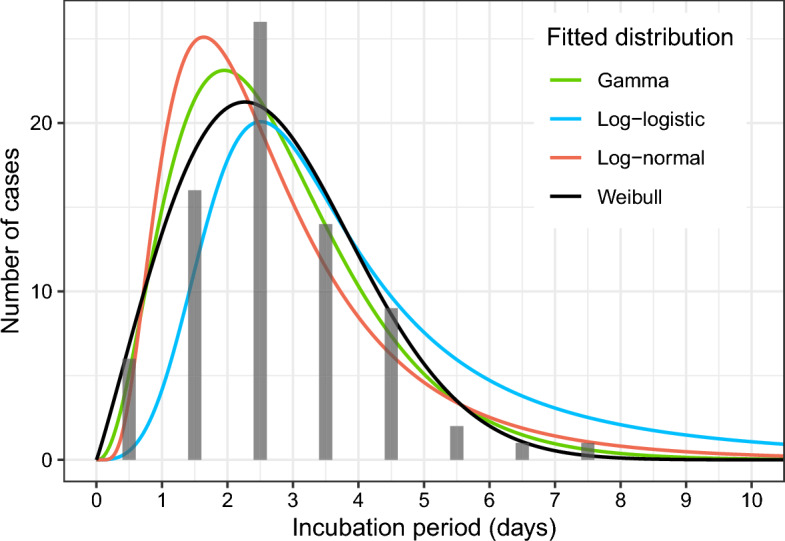
Observed incubation periods of 75 cases possibly or likely infected with the Omicron BA.1 strain on NYE. Various non-negative distributions are fitted using the maximum-likelihood method. The best fit is obtained with a Weibull distribution (shape and scale parameters 2.08 and 3.11, respectively) with a median of 2.6 days.

An assumption that source investigation accurately pinpointed the moment of infection on NYE allowed us to assess the distribution of incubation periods in this age group. We assumed that the infection occurred at midnight on NYE, which is the midpoint of our definition of NYE events and culturally is also the moment of closest interaction, as attendees hug and kiss to celebrate the new year. The median incubation period of Omicron was 2 to 3 days, when considering all symptomatic cases possibly or likely infected on NYE (mean: 2.8 and 2.4 days, N: 75 and 44, respectively). After fitting several distributions, we attained the best fit for possible transmissions on NYE with a Weibull distribution (shape and scale parameters k = 2.08 and λ = 3.11), with a median of 2.6 days (5th and 95th percentile 0.7 and 5.3 days, respectively) *(*Fig. 3*).*

### National reproduction number

**Figure 4:**
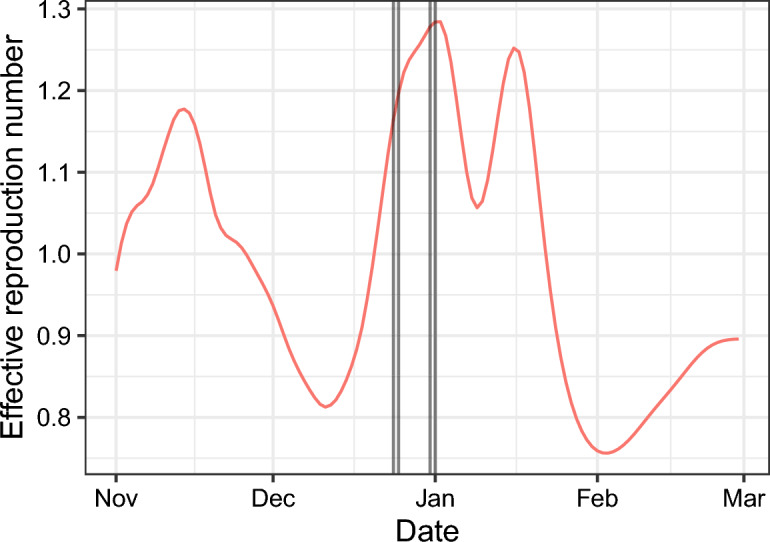
Estimate of the effective reproduction number (R_eff_) on a national scale. Reported case numbers in Belgium were used to estimate R_eff_ with EpiInvert. Vertical black lines indicate the following dates, which typically correspond with holiday celebrations: 24 December, 25 December, 31 December, and 1 January.

We visualise the effect of Christmas and NYE celebrations on a national scale by estimating R_eff_ from publicly available daily case numbers *(*Fig. 4*)*^20^. As symptom onset data were not available, we corrected for weekly reporting effects using the method by Alvarez *et al*^21^. This method, which results in smoothing of the estimated reproduction number, shows a wide peak around Christmas and NYE. A second peak follows in the first week of schools reopening.

Similar peaks in transmissivity around the holiday celebrations can be observed in many surrounding European countries (Supplementary Fig. 3).

## Discussion

Taken together, these results suggest a strong link between simultaneous social gatherings on NYE and a subsequent surge of detected COVID-19 cases amongst students in Leuven, Belgium. The combined phylogenetic and contact tracing data imply that small-scale social gatherings on NYE, rather than superspreading events, were the main contributor to a surge in new cases.

Mandated restrictions on social interactions resulted in limited event sizes, taking place mainly in private residential settings. As a result, despite a high secondary attack rate, superspreading events were responsible for only 16 to 34% of cases infected on NYE. Both contact tracing and phylogenetic data indicate that the surge of infections was not caused by one or a few superspreading events.

To our knowledge, this study shows the first example in the literature of a strong link between public holiday celebrations and a surge in cases of SARS-CoV-2. Interestingly, simultaneous celebrations in small circles, rather than mass gatherings, appeared as the main driver of the surge.

The observed wave of cases consisted largely of first-generation infections directly originating from these social gatherings. Incidence in the targeted student population quickly stabilised thereafter, likely due to a combination of government-mandated contact restrictions, low threshold testing, contact tracing and reduced social interaction due to the students’ approaching January exams.

However, in the absence of these limiting factors, such a simultaneous increase in transmissivity could logically accelerate an exponential rise in cases. In fact, nationally reported case numbers and their derived effective reproduction numbers suggest that this may be exactly what happened on a national and even international scale with the spread of the Omicron BA.1 strain.

Estimates of the national effective reproduction number suggest a possible “triple whammy” of transmissibility effects around the winter holidays. First, Christmas and New Year celebrations provide two occasions for large scale inter-generational and inter-regional transmission, as friends and family gather with a range of risk factors: enclosed spaces, extended contact, close physical interaction, inter-household contact and strong vocalisation. The resulting cases have relatively little opportunity for onward transmission until schools and many workplaces reopen simultaneously, leading to further transmissions within regions and generations. Further research might confirm the relative contributions of transmission across regions and generations.

The incubation period is an important parameter for epidemiological modelling. We attain similar values for the mean and median incubation period of the Omicron strain (2.7 and 2.6 days, respectively) as reported previously, while providing further data on the incubation period distribution^14,16^. Values ranged from less than one to eight days and followed a broad unimodal skewed-right distribution. The best fit was obtained with a Weibull distribution with 5th and 95th percentiles of 0.7 and 5.3 days, respectively *(*Fig. 3*).*

This study has several limitations. First, its focus on a population of higher education students limits generalisability to other age and socio-economic groups.

Second, determining the source event of an infection through contact tracing has inherent limitations. Our definition of “likely infected on NYE” excludes cases who reported multiple plausible source events, but self-reporting bias may still reduce the reliability of the data. Indeed, the phylogenetic analysis indicated that some cases linked to the same event were not closely related. This observation could further support a limited role of superspreading, but could also indicate that the NYE event was not the source of their infection. However, the association with NYE is further supported by a peak in the effective reproduction number and a surge in cases above the forecast.

Third, the study design and the lack of exposure data from negative controls preclude definite evidence for a causal link in the epidemiological sense.

Finally, we were unable to determine the number of secondary infections outside of the target population, and thus the direct effect of NYE on national case numbers.

In summary, a simultaneous increase in social interactions, as can occur during holiday celebrations, can lead to a surge of SARS-CoV-2 infections. This can happen even in the presence of social restrictions, through an increase of transmissions at small-scale gatherings.

## Methods

### Setting

The study was conducted in the setting of a dedicated testing and contact tracing program targeting around 50,000 higher education students at KU Leuven Association in Leuven, Belgium. A test and trace program was implemented, specifically targeting this student population. Students could attend for a PCR test at no cost and even in the absence of specific test criteria. The test centre closely cooperated with the contact tracing team to improve the flow of information.

New Year’s Eve 2022 (NYE) encouraged many students to briefly meet with friends and family, before returning to a generally solitary period of studying for their January tests. On a national level, the Omicron BA.1 strain was dominant and rising at the time^22^. Government-mandated restrictions resulted in limited event sizes *(*Supplementary Fig. 2*)*^23^.

### Inclusion and exclusion

In the days after NYE, we observed a surge in case numbers and initiated further investigation. We attempted to interview each case with a positive result on the TaqPath COVID-19 assay at the university test centre between 25 December 2021 and 14 January 2022. Cases with a previous positive test result in the 60 days preceding their sample collection were excluded, as well as samples with only a weak positive signal (C_q_ value for SARS-CoV-2 N-gene target above 30). To construct the epidemic curve, we plotted symptomatic cases according to self-reported disease onset date. Based on national genomic monitoring, S-gene target failure of the TaqPath COVID-19 assay was used as a marker of the Omicron BA.1 lineage^22^. S-gene positive samples were considered as Delta before and Omicron BA.2 from 17 January 2022 onwards, corresponding to the first week when the share of new Omicron BA.2 cases overtook the share of Delta cases on a national level *(*Supplementary Fig. 1*)*^22^.

### Effective reproduction number

For the local student population, R_eff_ was estimated from the epidemic curve as described by Cori et al*.* with a one-day sliding window^17^. A one-day window was expected to result in broad credible intervals. However, this window was chosen because we were investigating a suspected change in transmissibility occurring during a very short time (NYE). Therefore, estimates of daily variability were considered useful. We used a parametric gamma distribution of the serial interval with a mean of 2.75 days (SD: 2.54 days)^24^. A Grubbs test was used to determine whether the main peak of the R_eff_ estimate was an outlier or a stochastic effect.

We inferred R_eff_ on a national scale from publicly reported case numbers^20^. As their symptom onset dates were not available to us, we inferred R_eff_ from sampling dates using a 7-day sliding window in the R package EpiInvert. This method corrects for reporting biases such as the “weekend effect” but smooths the estimated value of R_eff_^21^. We did not use the option to ignore the effect of public holidays on R_eff_, because they are the subject of this study. The serial interval was assumed normally distributed with a mean of 2.75 days (SD: 2.54 days). We did not correct for delays between symptom onset and sampling.

### Forecast

To construct a forecast of symptomatic case numbers amongst students, we first estimated R_eff_ with the above method (Cori et al., one-day window). However, here we only included cases with symptom onset before NYE. We used the mean R_eff_ estimate in the seven days before NYE to calculate the forecast, assuming that R_eff_ was constant throughout this period.

### Definition of superspreading event

A SSE was defined as an event with more transmissions than would originate from 99% of infectious cases, when assuming a simple Poisson distribution of infections^25^. Using our estimated R_eff_ of 3.6 for Leuven students on NYE, events with at least nine transmissions were considered superspreading events*.*

An alternative would have been to base our definition of a SSE on the estimated R_eff_ in the days before NYE. However, in that case, any increase in transmissibility on NYE – even homogenously distributed across the population – would have resulted in a higher observed proportion of transmissions occurring at superspreading events. Our aim was instead to determine whether individual variation in transmissibility (i.e., superspreading) increased on NYE. Therefore, in our definition of a SSE, the threshold number of transmissions varies with R_eff_.

### Source investigation

At least one attempt was made to contact each student with a positive PCR test for a telephone interview, in an effort to trace the source of their infection and their close contacts during the contagious period. Close contact was defined as direct contact, or an interaction with at least 2 of the following characteristics: duration longer than 15 min, distance shorter than 1.5 m and absence of face masks. The contact elicitation window was defined as starting 7 days before diagnosis or symptom onset of the case, whichever was earlier^26^.

Particular attention was paid to NYE events, even if this meant extending the contact elicitation window. A NYE event was defined as any social gathering involving close contact (within or outside the household) occurring between noon on 31 December 2021 and noon on 1 January 2022. The contact tracers determined which cases were possibly and likely infected on NYE using the following criteria. Cases were classed as “possibly infected on NYE” if they had attended an NYE event (with or without other known cases in attendance), developed symptoms 0 to 14 days afterward, and – in the period from 1 week before NYE to symptom onset – had no close contact with a potential source case who was not present at the NYE event. Cases fulfilling these criteria were additionally considered “likely infected on NYE” if they were in contact with another known case during the NYE event, but not on any of the preceding or subsequent seven days.

### Phylogenetic analysis

Our phylogenetic analysis included, first of all, the genomic sequences obtained from students possibly or likely infected on NYE.

In addition, we included all Belgian Omicron sequences available on GISAID, sampled between 1 November 2021 and 20 January 2022, and analysed for the purpose of baseline genomic surveillance. This was determined by searching for the word “baseline” in the “sampling strategy” metadata field. If this field was blank, the sequence was excluded.

Finally, a selection was included of other European Omicron sequences from the same period, labelled as “high coverage” by GISAID. “High coverage” here indicates less than 1% of undefined bases, less than 0.05% unique amino acid mutations, and no insertions or deletions unless verified by the submitter. The selection was obtained through proximity sampling – genetic similarity to the included Belgian sequences – and limited to 20 genomic sequences per country and per month.

Sequences with fewer than 27,000 known bases or not belonging to the BA.1 lineage were excluded.

The Nextstrain pipeline with its default parameters was used to construct a time-scaled phylogenetic tree^27^. Multiple sequence alignment was accomplished using MAFFT, with the WIV04 genome (GISAID accession number EPI_ISL_402124) as the reference sequence^28^. A phylogenetic tree was constructed using Augur, calling IQ-TREE with a general time reversible model, and time-scaled using TreeTime^29–31^. Phylogenetic clustering was performed with the cov2clusters method, using previously reported coefficients (ß_0_ = 3, ß_1_ = − 19,735.98, ß_2_ = − 0.075) and a pairwise probability threshold of 90%^19^.

As our analysis was focused on the Omicron BA.1 lineage, only the corresponding Nextstrain clade (21 K) was visualised using the R package ggtree^32^.

### Ethical approval

The study protocol was approved by the Ethics Committee Research UZ / KU Leuven (reference no. S64919). The planning, conduct and reporting of the study were performed in line with the Declaration of Helsinki, as revised in 2013. The Ethics Committee Research UZ / KU Leuven waived the need for individual informed consent, since the data gathered did not exceed what was required for the purpose of safeguarding public health.

### Supplementary Information

Supplementary Figures.

Supplementary Tables.

## Data Availability

The findings of this study are based on metadata associated with 8,770 sequences available on GISAID up to 10 October 2022, via gisaid.org/EPI_SET_221010sa (NYE samples, Supplementary Table 4) and gisaid.org/EPI_SET_221128mt (Belgian and European context, Supplementary Table 3). All other data generated or analysed during this study are included in this published article and its supplementary information files (Supplementary Tables 1, 2).
