# Supplementary material for: Unravelling the effect of New Year’s Eve celebrations on SARS-CoV-2 transmission
Source: Sci Rep. 2023 Dec 14;13:22195. doi: 10.1038/s41598-023-49678-x (PMC10721646; doi:10.1038/s41598-023-49678-x)
Supplement: Supplementary file 1 — Supplementary Figures. [file 41598_2023_49678_MOESM1_ESM.pdf]

Unravelling the effect of New Year’s Eve celebrations on SARS-CoV-2 transmission

Authors: Caspar Geenen, Jonathan Thibaut, Lies Laenen, Joren Raymenants, Lize Cuypers, Piet Maes, Simon Dellicour, Emmanuel André

Supplementary Figures

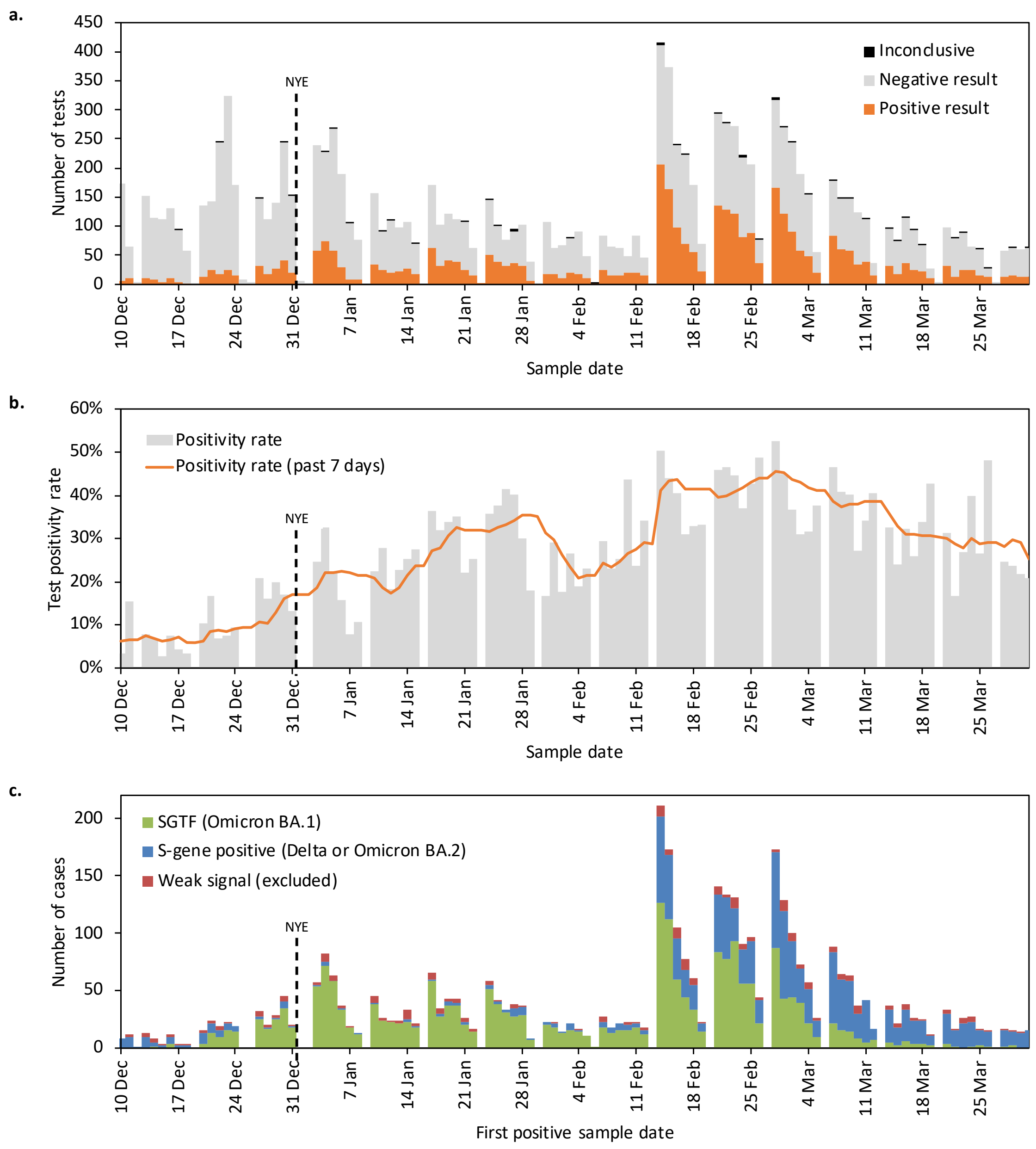

Supplementary figure 1. Results of all real-time reverse transcription PCR tests sampled at the KU Leuven test centre from 10 December 2021 to 31 March 2022. Panels a and b show the test results and the test positivity rates, respectively. Panel c shows the results of the S-gene target PCR for all initial positive test results. Based on national genomic monitoring, the S-gene target result of the TaqPath COVID-19 assay was used as an indicator of variants of concern. S-gene target failure was assumed to indicate the Omicron BA.1 strain. A positive S-gene result was labelled as Delta until 16th January 2022 and as Omicron BA.2 thereafter.

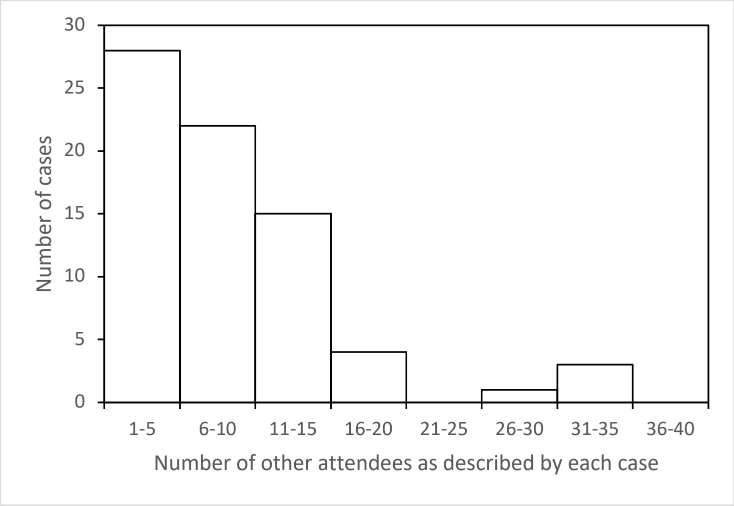

Supplementary figure 2. Event sizes on New Year's Eve. Each case likely or possibly infected on NYE was asked about the size of the gathering they attended. The figure excludes 17 cases who did not share this information in the interview. Event sizes were generally limited, with no cases reporting events larger than 35 attendees.

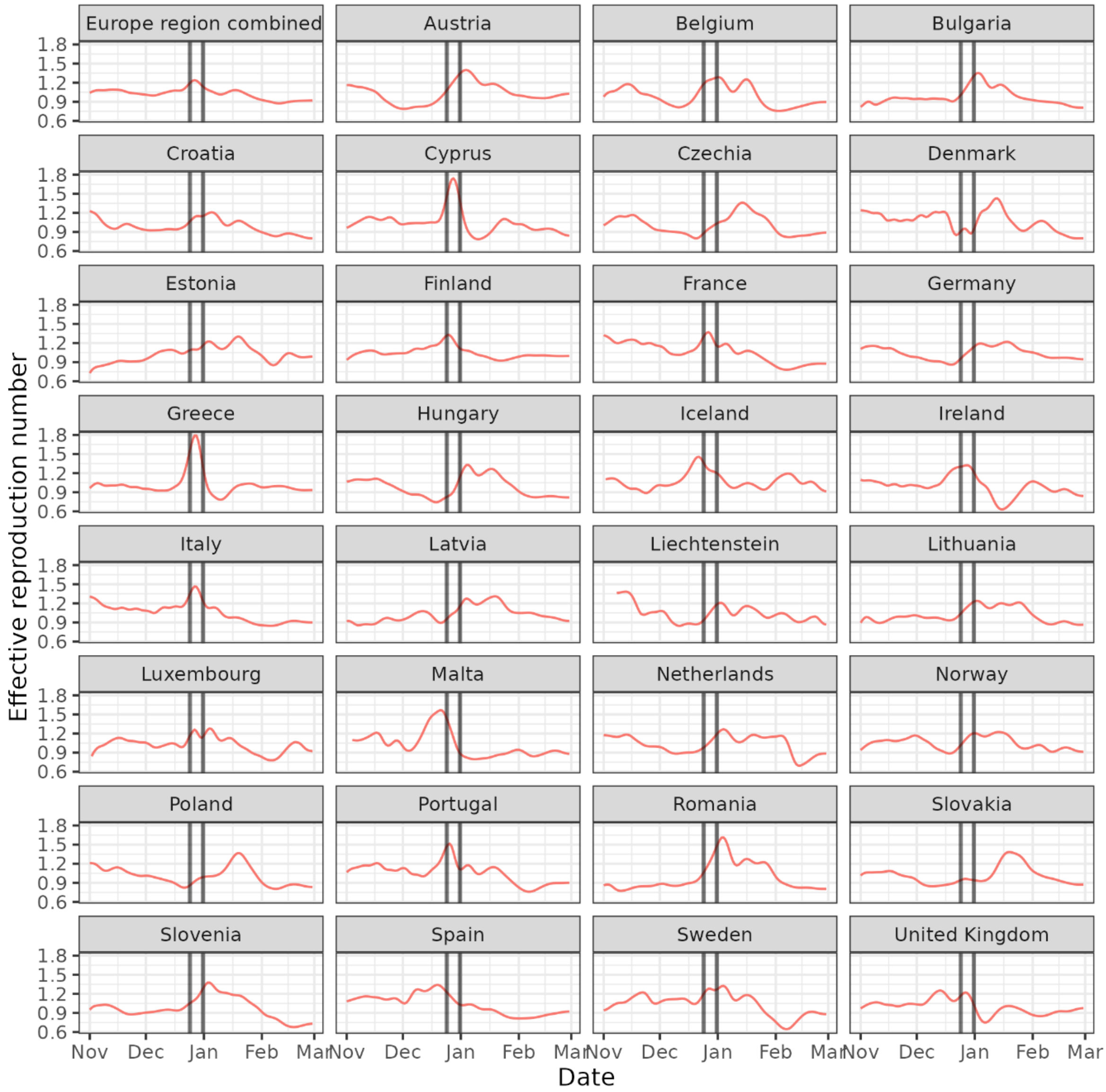

Supplementary figure 3. The effective reproduction number as estimated using EpiInvert from nationally reported case numbers[1,2]. Vertical lines indicate the following dates of interest: 24 December, 25 December, 31 December, and 1 January.

a.

b.

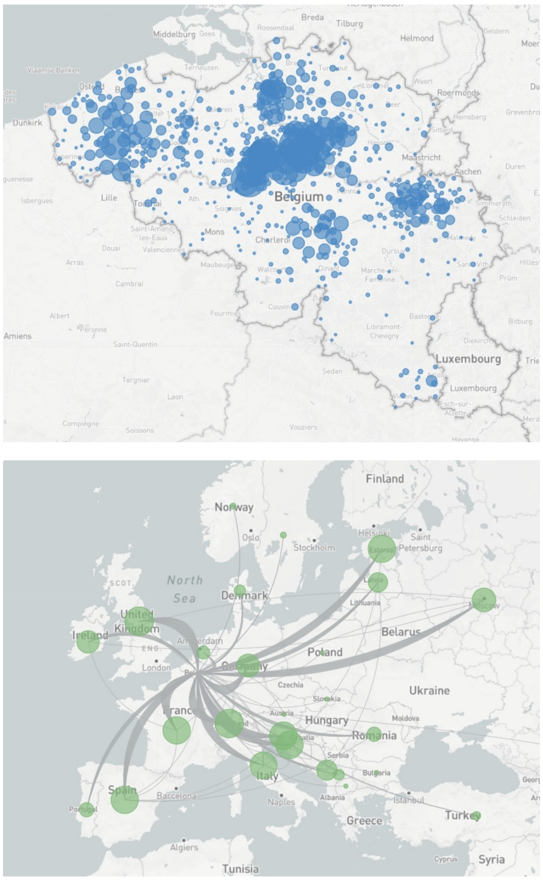

Supplementary figure 4. Geographic spread of sequences included in the phylogenetic analysis, generated in Auspice[3]. The maps show Belgian baseline surveillance cases (panel a, by postcode) and a selection of cases from other European countries (panel b, by country).

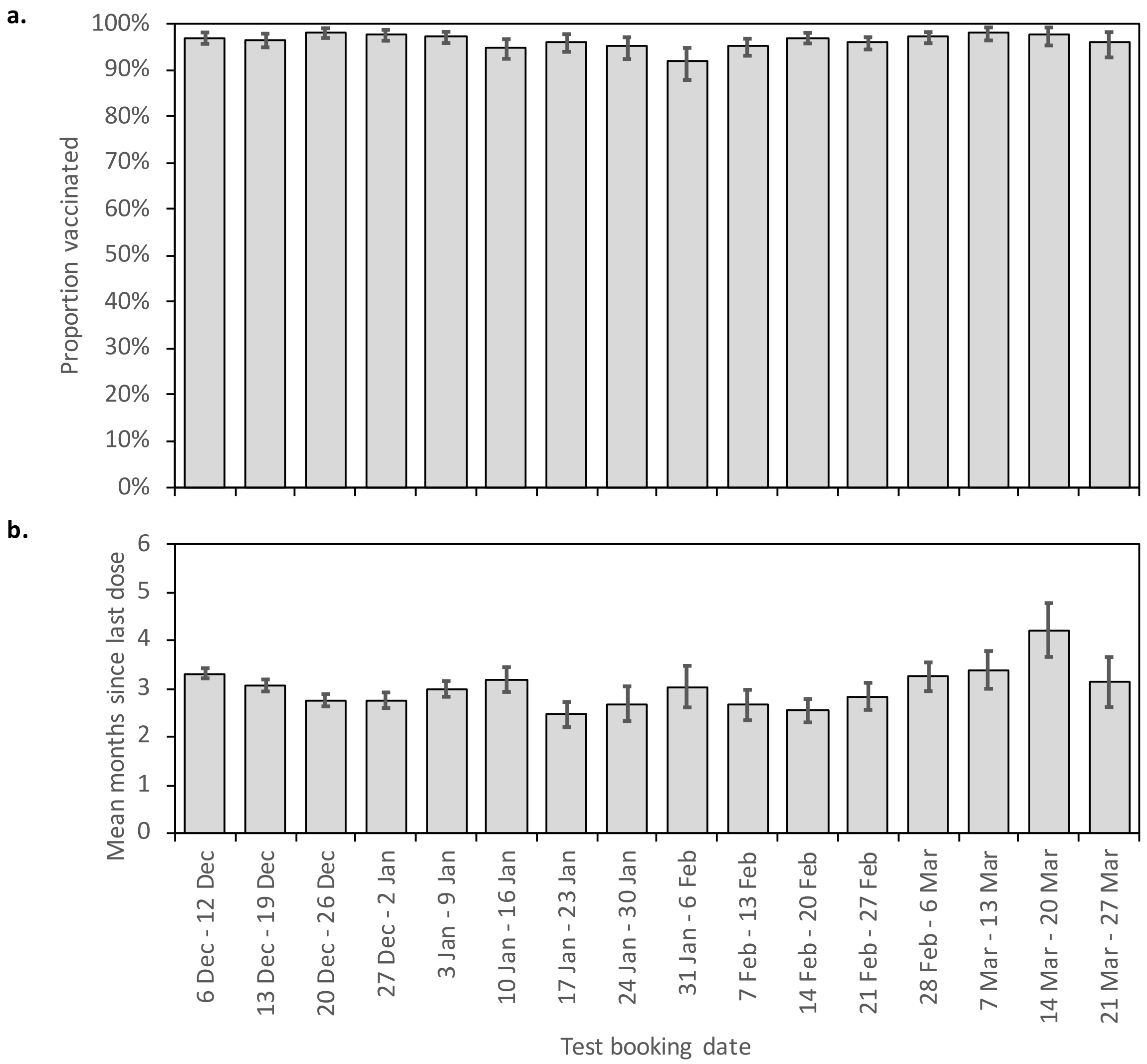

Supplementary figure 5. Vaccination status self-reported by attendees of the university test centre. Each student booking a test in the university test centre was asked in an online form whether they had been vaccinated for COVID-19, and if so, the month of their last dose. Vaccination rates are shown in panel a, while panel b shows the mean number of months since the last vaccination dose. Error bars indicate Clopper-Pearson and t-based 95% confidence intervals, respectively.

References

1. Alvarez, L., Colom, M., Morel, J.-D. & Morel, J.-M. Computing the daily reproduction number of COVID-19 by inverting the renewal equation using a variational technique. *Proceedings of the National Academy of Sciences* **118**, (2021).

2. European Centre for Disease Prevention and Control. Data on the daily number of new reported COVID-19 cases and deaths by EU/EEA country. https://www.ecdc.europa.eu/en/publications-data/data-daily-new-cases-covid-19-eueea-country.

3. Hadfield, J. *et al.* Nextstrain: real-time tracking of pathogen evolution. *Bioinformatics* **34**, 4121–4123 (2018).
